# Analysis of insulin glulisine at the molecular level by X-ray crystallography and biophysical techniques

**DOI:** 10.1038/s41598-021-81251-2

**Published:** 2021-01-18

**Authors:** Richard B. Gillis, Hodaya V. Solomon, Lata Govada, Neil J. Oldham, Vlad Dinu, Shahwar Imran Jiwani, Philemon Gyasi-Antwi, Frank Coffey, Andy Meal, Paul S. Morgan, Stephen E. Harding, John R. Helliwell, Naomi E. Chayen, Gary G. Adams

**Affiliations:** 1 Faculty of Medicine and Health Sciences, Queen’s Medical Centre, University of Nottingham, Nottingham, NG7 2HA UK; 2 Division of Systems Medicine, Department of Metabolism, Digestion and Reproduction, Faculty of Medicine, Imperial College London, Sir Alexander Fleming Building, London, SW7 2AZ UK; 3 School of Chemistry, University of Nottingham, University Park, Nottingham, NG7 2RD UK; 4 National Centre for Macromolecular Hydrodynamics, School of Biosciences, University of Nottingham, Sutton Bonington Campus, Loughborough, LE12 5RD UK; 5 Universitetet I Oslo, St. Olavs plass, Postboks 6762, 0130 Oslo, Norway; 6 Department of Chemistry, University of Manchester, Manchester, M13 9PL UK

**Keywords:** Biophysical chemistry, Molecular conformation, Diabetes, Mass spectrometry, X-ray crystallography

## Abstract

This study concerns glulisine, a rapid-acting insulin analogue that plays a fundamental role in diabetes management. We have applied a combination of methods namely X-ray crystallography, and biophysical characterisation to provide a detailed insight into the structure and function of glulisine. X-ray data provided structural information to a resolution of 1.26 Å. Crystals belonged to the H3 space group with hexagonal (centred trigonal) cell dimensions a = b = 82.44 and c = 33.65 Å with two molecules in the asymmetric unit. A unique position of D21Glu, not present in other fast-acting analogues, pointing inwards rather than to the outside surface was observed. This reduces interactions with neighbouring molecules thereby increasing preference of the dimer form. Sedimentation velocity/equilibrium studies revealed a trinary system of dimers and hexamers/dihexamers in dynamic equilibrium. This new information may lead to better understanding of the pharmacokinetic and pharmacodynamic behaviour of glulisine which might aid in improving formulation regarding its fast-acting role and reducing side effects of this drug.

## Introduction

Insulin glulisine (IGlu) is a rapid-acting insulin analogue, which differs from human insulin, where the asparagine at position B3 is replaced by lysine and lysine in position B29 is replaced by glutamic acid. Chemically, it is 3B-lysine-29B-glutamic acid-human insulin, which, when injected subcutaneously, the onset of action is more rapid, and achieves higher concentrations compared to human insulin on a unit-per-unit basis. IGlu is an analogue of human insulin, which affects self-association (into dimers) and the isoelectric point, shifting to a low pI 5.1 compared to human insulin, pI 5.5, which augments its solubility at a physiological pH. IGlu, like human insulin, is formed of two chains, stabilised with two inter-chain, and one intra-chain, disulfide bridges, commonly referred to as A and B^1^.

IGlu, the recombinant human insulin analogue, has been reported to reduce the propensity to form hexamers (a stable, but ineffective form) and remain as monomers (the effective form), thereby making it a fast-acting analogue^2^. IGlu takes effect 12–30 min after injection and peaks at 1.6–2.8 h, as opposed to human recombinant insulin which takes 30 min to take effect and peaks at between 2.5–5 h^3^.

Although several characterisation studies have been published on IGlu^4–9^, its crystal structure has not been reported in detail. We have obtained crystals^10^ and in this elucidation study, we identify for the first time, the results of crystallography combined with data from analytical ultracentrifugation to provide structural information that was previously lacking. The combination of methods provides a detailed and comprehensive insight into the structure and function of the IGlu, which can assist in designing an improved formulation as a rapid-acting insulin and reducing side effects of this drug.

## Results and discussion

### X-ray crystallography

The presence and integrity of the IGlu sample was confirmed by Mass Spectrometry (Fig. S1, results and methods are shown in Supplementary Information). The atomic structure of IGlu was then determined by crystallisation and X-ray diffraction (PDB code 6GV0).

### Crystals

Crystals grew after 24 h with typical dimensions of 0.2 × 0.2 × 0.2 mm (Figure S2). Following detailed investigations of the crystallisation phase diagram^10^, the best diffracting crystals up to 1.26 Å resulted from mixing 1 µL of the protein solution with 1 µL of the crystallisation reservoir solution containing: 0.2 M Mg-Formate, 0.1 M Bis–Tris buffer pH 5.5 and 8.0% glycerol.

The X-ray diffraction data demonstrates that the IGlu crystal belongs to the H3 space group with hexagonal (centred trigonal) cell dimensions a = b = 82.44 Å and c = 33.65 Å, and angles 90, 90, 120 degrees with two molecules in the asymmetric unit cell (we refer below to Mol1 consisting of chain A and chain B and Mol2 consisting of chains C and D).

Positive and negative electron density around the side chains of residue B3 (in Mol1 and Mol2) confirmed that indeed this residue was mutated from Asn to Lys. As expected, D3 has electron density which is stronger than B3. As for the second mutation, Lys to Glu at position B29 the B29 mutation in Mol2 (which we label as D29) mutation was observed while in Mol1 it is not since the C-terminus of the B chain B29 and B30 (from B29 Cβ) is disordered.

### Model building

Strong electron density (> 6σ in Fo–Fc maps) was visible at the known Zn^2+^ site and was therefore modelled as such. The final model of the IGlu complex consists of 76 water molecules, two Zn^2+^ ions—one for each monomer (shared with two other symmetry related molecules) and two formate ions. Ten amino acid residues were modelled in alternate conformations for the dimer in which some alternates were introduced into relatively low density of 0.7–8σ due to flexibility of the residues. The C-terminus of chains B and D (long chains of insulin monomers) also shows some flexibility around residues B29 and the last residue Thr30 of both chains (B30 and D30) could not be modelled into electron density.

Overall parameters for the diffraction statistics and the final model are listed in Table 1. A representative section of the final electron density map is shown in Figure S3a.

**Table 1:** Data collection and processing parameters of glulisine.

Beamline	Diamond-I04-1
Δφ (°)	0.2
φ_0_ (°), total frames	180, 900
Rmerge (last shell) %	5.8 (69.1)
$\left\langle {{\text{I}}/\sigma ({\text{I}})} \right\rangle$ (last shell)	13.4 (1.2)
Completeness (last shell) %	99.8 (99.3)
Space group	H3
**Unit cell**	
a = b (Å)a = b (Å)	82.44
c (Å)	33.65
α = β (°)	90
γ (°)	120
Resolution (Å)	41.22–1.26
Rf	0.1279
Rfree	0.1518
**Ramachandran (%)**	
FAVORED	97.44
ALLOWED	1.28
OUTLIERS	1.28
Number of Zn ions	2
Number of formate ions	2
Number of water molecules	76
Angle r.m.s.d	1.887
Bond length r.m.s.d	0.023
Average B-factor Å^2^	20.5
PDB code	6GV0

### The overall structure of the glulisine monomer (dimer)

**Figure 1:**
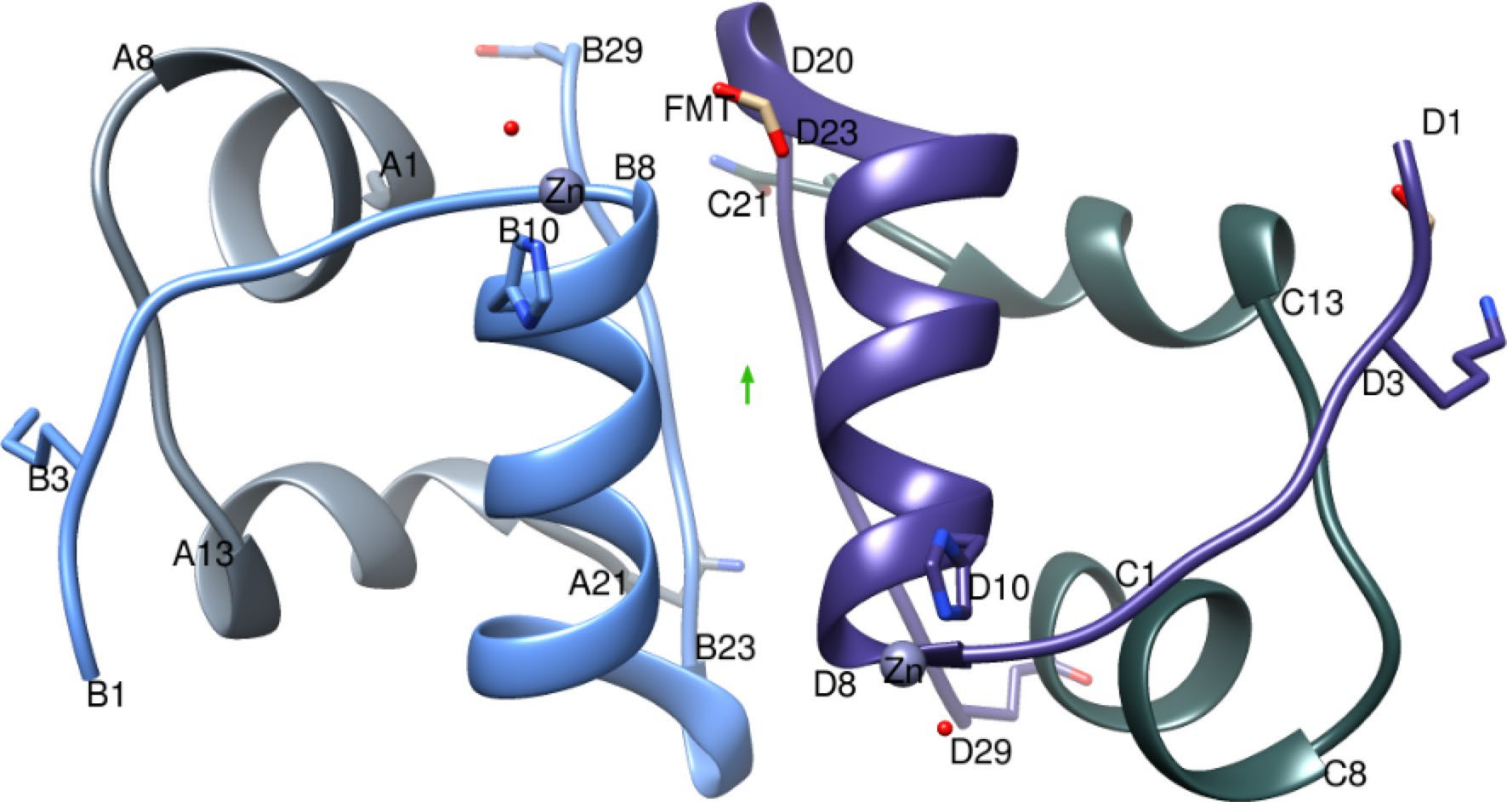
IGlu dimer with the two-fold like axis (green arrow), A-chain is coloured light grey, B-chain in light blue, C-chain in dark grey and D-chain in purple. Figure created using Chimera v1.8.1 http://www.cgl.ucsf.edu/chimera/.

In the asymmetric unit of the IGlu crystal there are two independent molecules (a dimer): molecule1 (Mol1) comprising of peptide chains A and B and molecule2 (Mol2) comprising peptide chains C and D. Chains A and C are identical in sequence, as are chains B and D. The A-chain has 21 amino acids and the B-chain has 30 amino acids linked together by two disulfide bonds derived from cysteine residues (A7–B7 and A20–B19). In addition, there is one intrachain disulfide bond within the A-chain (A6–A11). The A-chain consists of two main α-helices A2Ile to A8Thr and A13Leu to A19Tyr connected by a non-canonical turn of A9Ser–A10Ile to stabilise the A6Cys–A11Cys disulfide bond. The B-chain of IGlu comprises a main α-helix (B8Gly to B19Cys) and an extended 3_10_ α-helix from B20Gly to B22Arg which allows the chain to fold back on itself bringing the B-chain C-terminal close to the N-terminus of A-chain^11^ (Fig. 1). The N-terminus of the B-chain of insulin may adopt two alternative conformations designated as the T- and R-states. The IGlu B-chain N-terminus is found in the T-state in the crystal. The T-state is where the B1Phe-B8Gly residues are in an extended chain while in the R-state the chain has undergone a conversion to an allosteric conformation thus extending the B-chain α-helix. T-like state is needed for folding efficiency and receptor binding^12^.

### The electron density and glulisine mutation

High quality electron density of the IGlu dimer was obtained and the model was well defined (Figure S3a). The following residues were found with good electron density in both of their split conformations: A12Ser, B9Ser, C12Ser, C18Asn and D27Thr while in residues B17Leu, D22Arg and B21Glu predominantly one alternate conformation could be modelled well. Thr30 of both B and D chains was not visible in the structure (see also PDB codes 4INS, 3ZS2, 1BE9). The C-terminal stretch of the B-chain insulin is known for its flexibility. This flexibility is important in insulin activation and self-association^13^.

**Figure 2:**
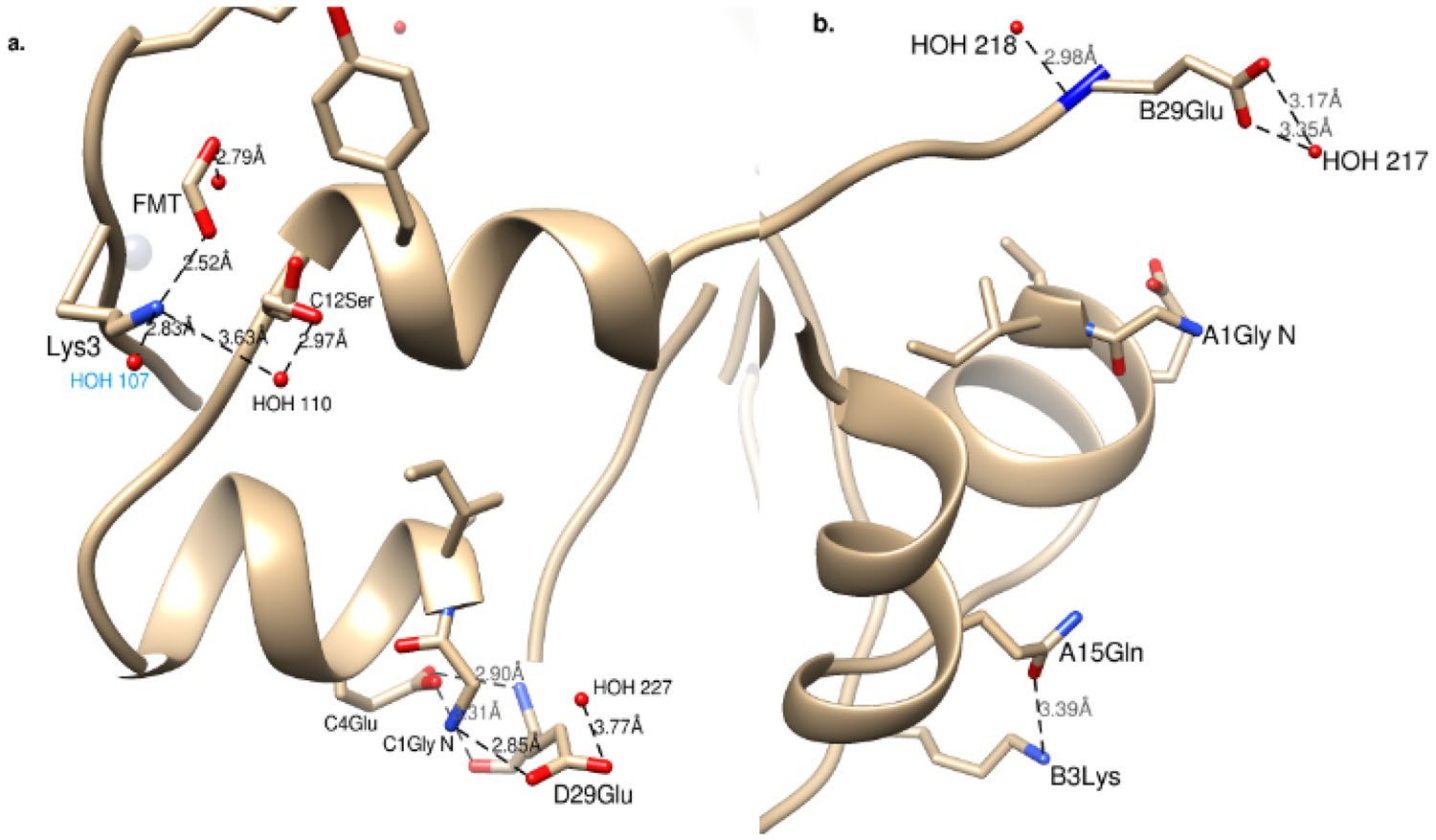
Hydrogen net bonds of the IGlu two mutations 3Lys and 29Glu for D-chain (**a**) and B-chain (**b**). Symmetry water 107 is labelled blue. Figure created using Chimera v1.8.1 http://www.cgl.ucsf.edu/chimera/.

IGlu differs from human insulin by replacing the amino acid asparagine at position B3 with lysine and the lysine at position B29 with glutamic acid. These two mutations are better fitted to the model at the D-chain of the dimer and all atoms are clearly seen at 0.9–1σ (Figures S3b,c). The B-chain is slightly more disordered thus only very weak density is observed for Lys3 NZ. For B29Glu no electron density is obtained from CB to CD. B3Lys NZ has only one weak hydrogen bond to the OE1 atom of A15Gln (3.39 Å) while D3Lys has 2 stronger hydrogen bonds, one to symmetry water 107 (2.83 Å) and the other to the O1 formate (FMT) ion (2.52 Å) (Fig. 2a). Both 3Lys are positioned at the surface of the molecule however, although chain B is closer to chain A compared with the N-terminal of the D-chain and C-chain, a FMT ion located in the gap between these chains enables better contact and thus more stability of the D3Lys side chain. D29Glu makes 2 H-bonds with the C-chain and one salt bridge: OE1 to N of A1Gly (2.85 Å), N to OE2 of A4Glu (2.90 Å) and carbonyl O to OE2 of A4Glu (3.31 Å) which enables clear observation of the 29Glu mutant (in the density) (Figure S3c, Fig. 2). B29Glu on the other hand has only H-bonds to water molecules (218 and 217), which can explain its weak electron density.

### Comparison to human insulin

IGlu structure alignment (superposition) with native human insulin (PDB codes 4EY9 and 4INS) shows a good overlap in general (for the root mean square deviations of specific chains calculated by PDBe fold see Table S1). The main differences observed are located at the C-termini of chains B and D. This is in agreement with the B29 mutation influencing the ability of the insulin IGlu for self-association as the B26Tyr-B30Ala region is important and may thus be more labile for dimerisation and hexamer formation. Smaller changes but notable ones, can be seen for the extended T region of these chains (residues 1–8) of the B chain but not (less for) the D.

### Comparison to human insulin dimers and hexamers

**Figure 3:**
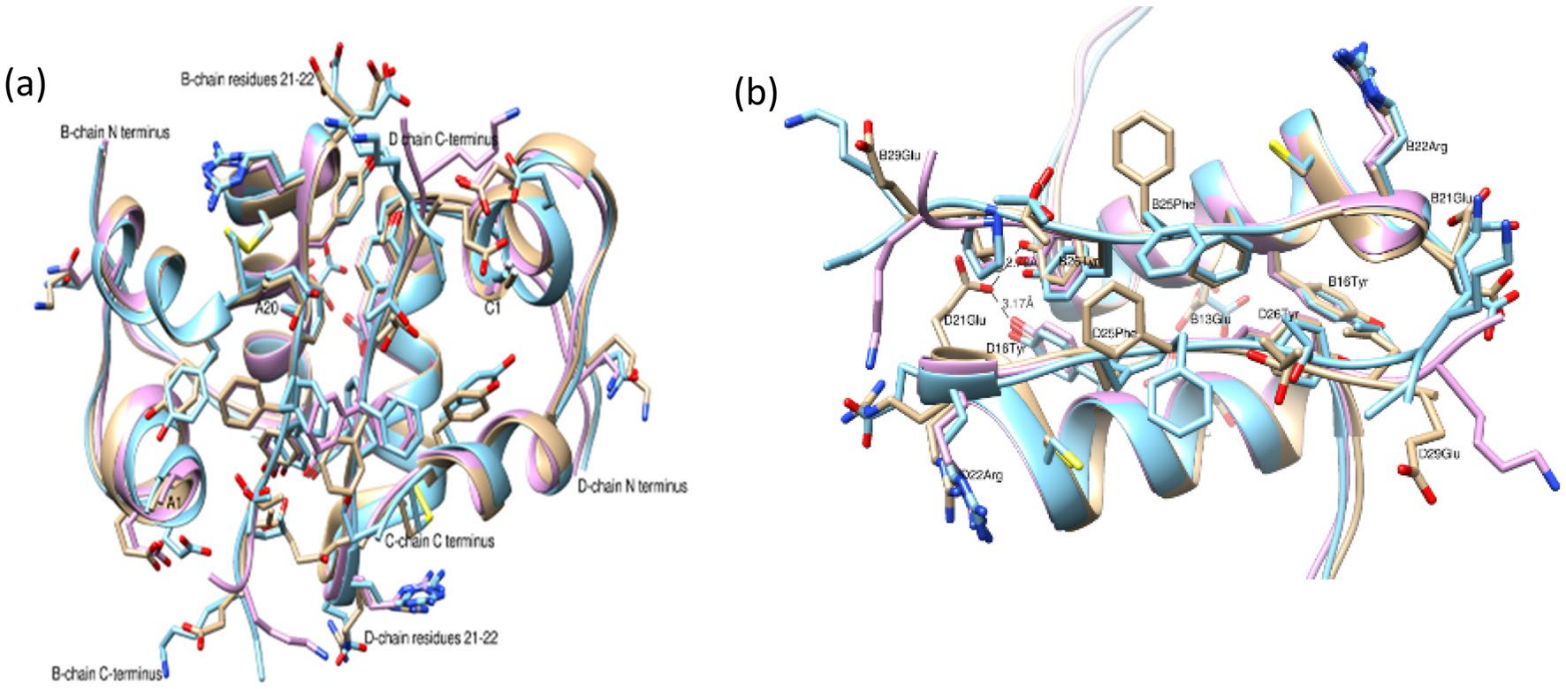
(**a**) The overall superposition of IGlu (tan) and human insulin 4INS (light blue) and 4EY9 (purple). Main differences are observed at the C-terminal of B/D chains and at D-chain residues 21–22. (**b**) A zoom along the two fold symmetry and the unique hydrogen bonds of D21Glu to B26Tyr and D16Tyr OH. Figure created using Chimera v1.8.1 http://www.cgl.ucsf.edu/chimera/.

**Figure 4:**
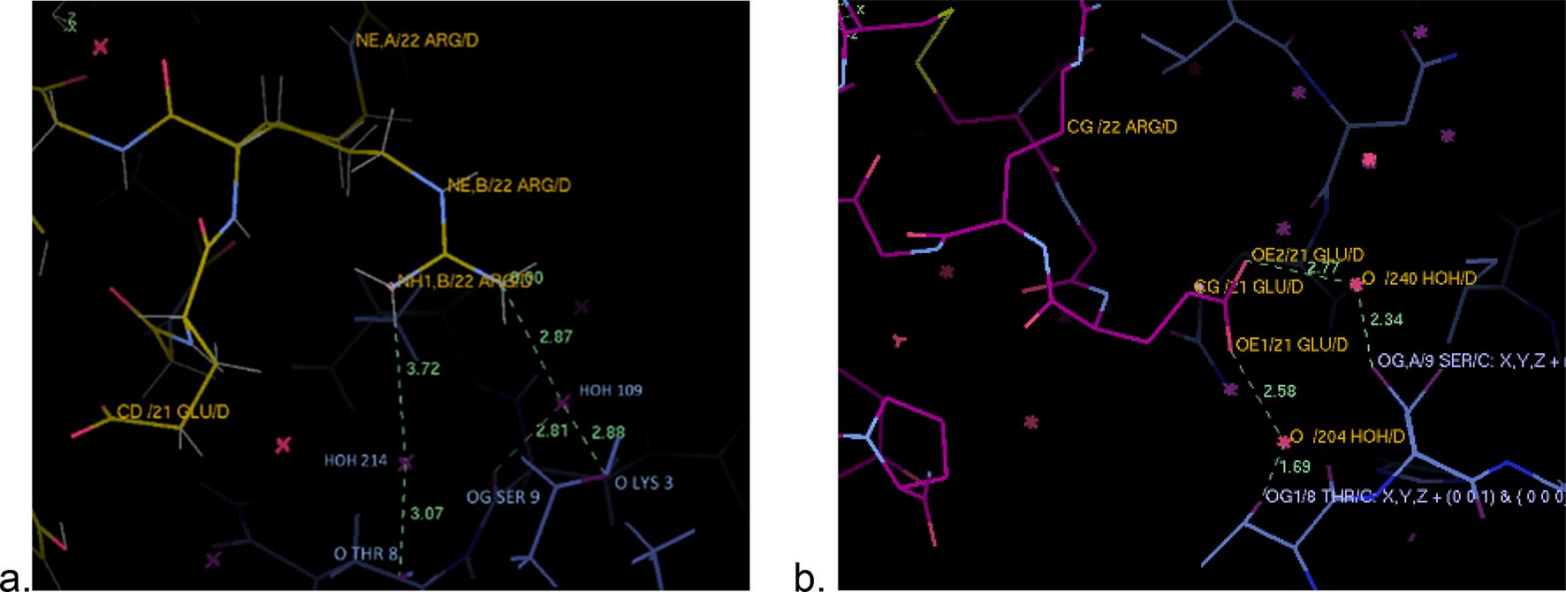
The unique conformation of IGlu D21Glu (**a**) and the native insulin D21Glu PDB:4EY9 (**b**) note their differing interactions through water molecules to a neighbouring protein molecule by symmetry. IGlu in yellow, insulin (4EY9) in pink and the symmetry related one in blue. Figures created using COOT v0.8.9.1.

It was found that residues B8Gly–B9Ser, B12Val–B13Glu, B16Tyr, B24Phe–B26Tyr and the C-terminal of the B chain are important for dimer creation and residues B1Phe–B7Cys B10His, B14Ala, B17Leu, B20Gly and A13Leu are essential for the hexamers. Superposition of IGlu, 4EY9 and 4INS show smaller changes for the D8–D12 residues compared with the B-chain (Fig. 3a). Nevertheless, IGlu D main chain is slightly diverted at D13Glu (about 0.6 Å distance) between the C atoms (same for N atoms) of IGlu and those of 4EY9 and 4INS. In addition, the D13Glu side chain is pointed to towards D10His, results in hydrogen bond to the N atom of D13His which is not found for D13Glu of the native insulin in 4INS and 4EY9. B13Glu is located at the centre of the hexamer, its side chain position is not conserved within the three mentioned structures. B13Glu in 4INS creates an H-bond with its approximate twofold crystallographic symmetry D13Glu (B13Glu OE2 distance to D13Glu OE2 is 2.59 Å) (Fig. 3b). Thus, the hexamer cavity is more closed. In insulin 4EY9, the side chain of B13Glu is located further away from the centre and thus makes hexamer cavity wider. Interestingly, the α-turn in the IGlu D chain residues D19Cys (from C atom), D20Gly, D21Glu and D22Arg is shifted away from the native insulin (4INS, 4EY9) bringing this region close to the B-chain pointing towards the inner core. This allows the creation of unique H-bonds between B26Tyr and D16Tyr OH groups to D21 OE2, while the human insulin D21Glu side chain points out to the surface of the dimer (towards the solvent) [PDB codes 4INS, 4EY9 (Figs. 3a and 4a), 4NIB, 4EWZ,1G7A]. This unique position of D21Glu was not observed for the two fast release insulin analogues Lispro and Aspart (PDB codes 5UPD and 4GBN accordingly, results are not shown). This position of D21Glu prevents the glutamic acid from creating a hydrogen bond to another molecule (by symmetry) therefore preferring the dimeric form. Although the D22Arg shows an alternate conformation in which one of them (the B) is located where the native D21Glu is, and may create contact to a symmetric molecule, the interaction would be very weak compared to the native Glu residue (Fig. 4). In addition, the electrostatic surface at this position is altered, as the negative charge of the Glu residue is hidden now. The last four labile residues at the C-terminal are probably influenced by the D29Glu (B29) mutation and its salt bridge to A1Gly N that contribute to stabilise the monomer^6^. However, this kind of bridge is observed for the insulin 4INS with its D30Ala O atom bonded to C1Gly N-terminus.

**Figure 5:**
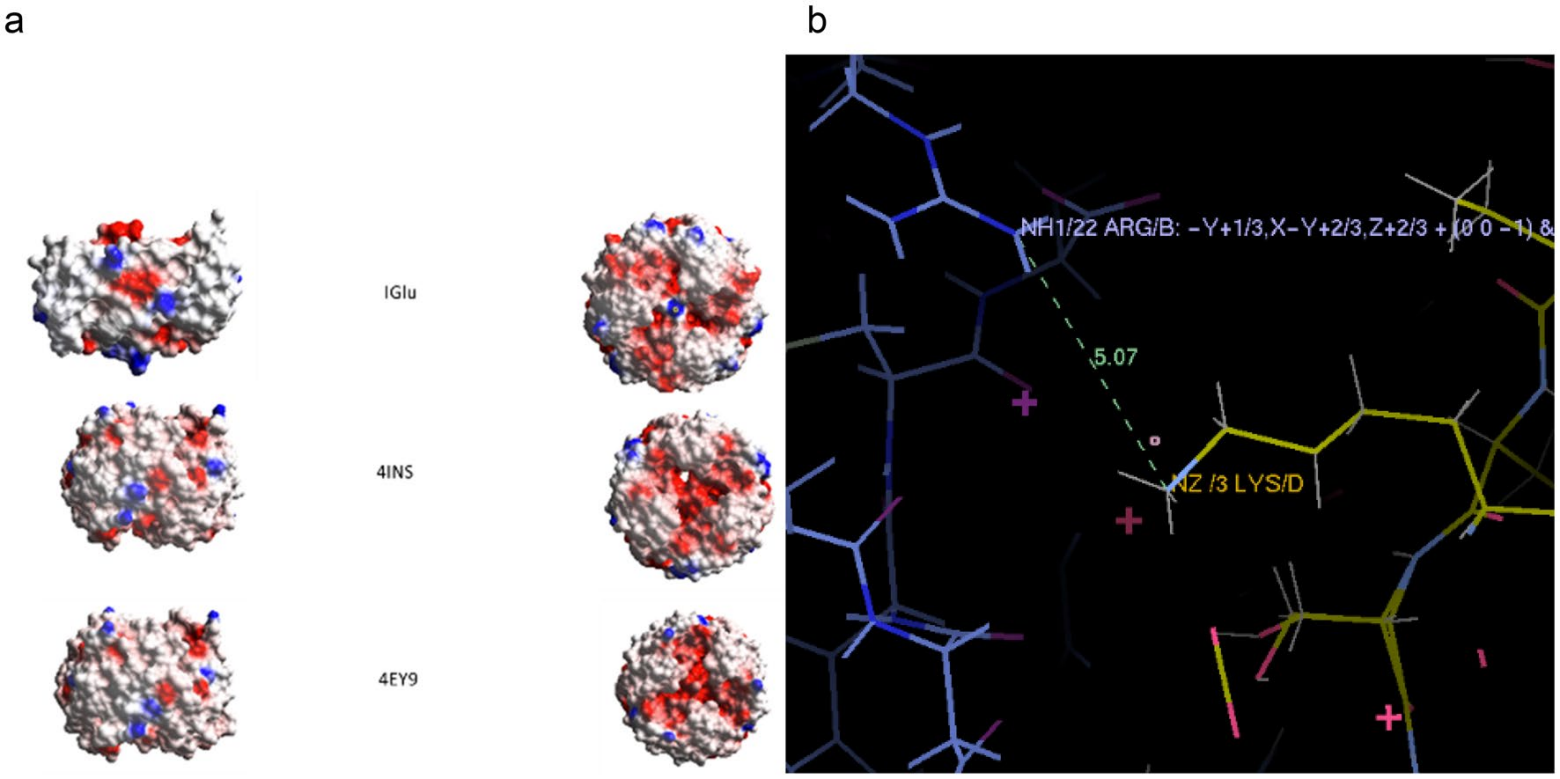
(**a**) The electrostatic surfaces of the hexamer of IGlu, 4INS and 4EY9 side view (left) and top view from the three fold axis (right). (**b**) D3Lys and B22Arg (symmetry) are both positively charged and in only 5.07 Å distance which contributes to the electrostatic repulsion. Figures created using COOT v0.8.9.1.

The electrostatic surface of IGlu is different than the native insulin because of the two mutations changing the neutral, polar, hydrophilic Aspargine at B3 to the basic, polar and hydrophilic lysine while at B29 the basic lysine is replaced with the acidic, polar and hydrophilic glutamic acid (Fig. 5a). The additional charge at B3 results in a slightly lower isoelectric point (pI) for IGlu of 5.1 compared with native human insulin 5.5. The B3Lys mutation also induced subtle steric and electrostatic repulsion between the two monomer molecules^6^. Figure 5b shows D3Lys in about 5 Å distance to a symmetry B22Arg that contribute to the electrostatic repulsion as both are positively charged (protein–protein electrostatic interactions carrying a charge can occur between molecules that are positioned up to 10 Å distance away from each other). Electrostatic surface calculations of IGlu, undertaken using COOT^14^ also indicate that B10His is basic while the electrostatic surfaces of 4INS and 4EY9 at the core of the hexamer are more acidic.

### The zincs, ligands, dimers and hexamers

The IGlu formulation is documented as a zinc-free insulin analogue for its rapid absorption action. Insulin crystallography has shown that zinc is pivotal for hexamer formation. With the B3 and B29 mutations disrupting the formation of dimers (B29) and hexamers (B3), IGlu is one of the best fast acting insulin derivatives^5,6^. However, our IGlu crystal structure consists of a dimer in the asymmetric unit (space group H3) that assembles to a hexamer. The differences between our IGlu and insulin are small rather than large differences, such as a different oligomerisation. The standard hexamer consists of six insulin molecules arranged as three dimeric units related by a threefold symmetry axis. The dimers possess a pseudo-twofold axis (some side chains have different arrangement thus breaking the twofold symmetry e.g. B/D25Phe side chain), which is perpendicular to the threefold axis of rotation (Figs. 1, 3b, Figure S4). The twofold symmetry axis is positioned between the antiparallel β-strands of B-chains. Strong positive electron density next to the B10His residue on the threefold axis was observed suggesting that a Zn ion should be modelled not least because this structural arrangement in insulin itself binds zinc. Indeed, the anomalous difference Fourier electron density map shows a strong peak at each of the two binding sites, which matches the classical Zn and with occupancy slightly less than the classical 1/3. We note that the X-ray wavelength of 0.92 Å is on the high energy side of the zinc K edge at 1.283 Å (i.e. f″ is 2.1 electrons at that X-ray wavelength). This finding must mean that traces of zinc ions are present in the commercial, as supplied, formulation solution as no zinc was used in the crystallisation.

We note that the commercial supplier does not document its sample with an X-ray fluorescence Scan. Interestingly, this Zn concentration presumably at a trace level, was sufficient for binding and for then ensuring hexamers in the crystal. However we also note that Gast et al.^5^ mentioned that IGlu forms compact hexamers even in the absence of Zn^2+^ and that in dilution the hexamers rapidly dissociate into monomers. In addition, they found that the IGlu association mechanism is different to the other rapid acting insulins. Insulin Aspart (NovoRapid) and Lispro (Humalog) (PDB codes 1ZEG and 1LPH, respectively) are both rapid-acting insulins and exist in hexameric form in their formulation and in the crystal structures in the presence of zinc but monomerisation (hexamer dissociation) is much more rapid in vivo compared to native insulin.

Two formate ions (FMT) from our IGlu crystallisation condition were observed in the electron density. The first FMT1 is located at a small pocket-like (D3Lys, C14Tyr, C12Ser, D1Phe) at the IGlu surface creating an H-bond with the basic D3Lys mutant. FMT3 is modelled in the middle of the hexamer along the threefold axis and creates an H-bond to the B10His ND1 atom (3.53 Å) from symmetry, which may have caused the His10 to be more positive as shown in the electrostatic surface. In both cases the FMT ions are located next to a basic residue.

The structure has now been thoroughly examined using X-ray crystallography. However, knowledge of the structure still does not provide information on how this analogue behaves in solution, and therefore its function in vivo. Hydrodynamic techniques were employed to elucidate this function.

### Analytical ultracentrifugation

#### Sedimentation velocity

**Figure 6:**
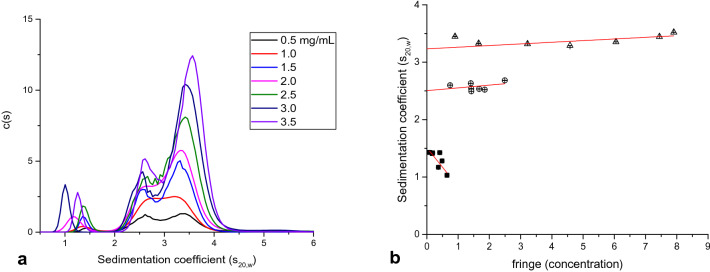
(**a**) Sedimentation coefficient profiles of IGlu at 7 concentrations between 0.5–3.5 mg/mL. (**b**) Integrated peaks plotted against concentration. Error bars are shown for both apparent sedimentation coefficient and concentration, however standard error was < 0.01% of values and therefore the error bars are indistinguishable from data points.

Figure 6a shows the c(s) distributions of a concentration series, ranging from 0.5–3.5 mg/mL, for IGlu. The distributions follow a consistent pattern of 3 peaks; a smaller, well-resolved peak at 1–1.5S and a pair of peaks between 2 and 4.5S. In lower concentrations (0.5, 1.0 mg/mL) these peaks are of equal height but as concentration increases, the height of the 3.5S peak increases more than the 2.5S peak. This is an indication of association between the two components. To confirm this, all three peaks were individually integrated for information on weight-average sedimentation coefficient and concentration (area-under-curve, fringe units). These results are plotted in Fig. 6b. Peak 1 shows an extrapolation to 1.5S at zero concentration from a negative slope, a classic example of non-ideal behaviour^15^. Peaks two and three, however, show positive slopes and extrapolate to 2.5S and 3.2S, respectively. The positive slope is confirmation of self-interaction, as the presence of more insulin in the system increased the proportion of higher molar mass stoichiometries.

The presence of an equilibrium of quaternary structures is evidence of the efficacy of IGlu as a fast-acting analogue. The complete dissociation into monomers functionalises the protein into the effective form, whereas the hexamer/di-hexamer conformations provide a more stable state when present in the sub-cutaneous depot.

#### HYDROPRO

HYDROPRO was used to estimate the sedimentation coefficients based on the previously obtained crystal structure. It estimated that the sedimentation coefficient of the dimer should have been 1.47S (based on molar mass of 11,646 Da and partial specific volume of 0.729 mL/g). This is in excellent agreement with peak 1 of the distribution. It can also be inferred that peaks two and three are the hexamer and dihexamer, respectively, as following a 2/3rds power scaling law, one would expect peaks of 2.7S for 35 kDa and 3.1S for 70 kDa. HYDROPRO also predicted an intrinsic viscosity of 3.822 mL/g, diffusion coefficient of 11.36 × 10^−7^ cm^2^/s, hydrated radius of 1.887 nm and radius of gyration of 1.476 nm. These properties were not measured as part of this investigation, however the diffusion coefficient is in agreement with previously obtained results^4^. The distribution, in terms of quaternary structure, is in disagreement with Nagel et al.^16^ who ascertained a system of monomers, hexamers and dihexamers, obtained through small angle X-ray scattering under similar conditions. It is possible there may be some error in sedimentation velocity measurement, related to the high proportion of larger oligomeric species, however the strong agreement with HYDROPRO does suggest that these results are valid. Alternatively, from the 1.5S peak showing strong concentration dependence and presenting as low as 1S at the highest concentration, could suggest some sort of complex monomer–dimer interaction which has not been elucidated by either study.

#### Sedimentation equilibrium

**Figure 7:**
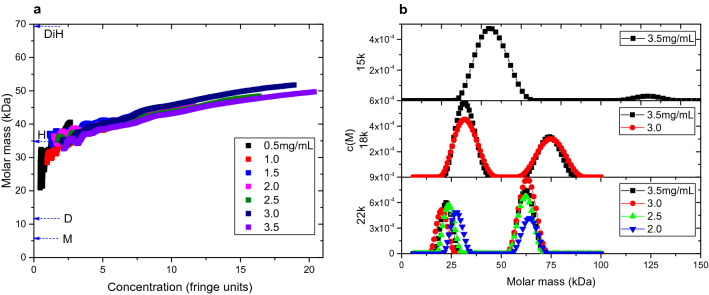
Output from SEDFIT-MSTAR c(M) analysis of IGlu measured with AUC-SE. (**a**) Point-average, apparent molar mass measured across the cell with overlapping plots from the concentration range 0.5–3.5 mg/mL). Monomer, dimer, hexamer and dihexamer have been labelled M, D, H and DiH, respectively. (**b**) c(M) distribution analysis from multi-speed experiments (15k, 18k, 22k revolutions per minute), where peaks were suitably resolved.

AUC-SE allowed for the determination of molar mass in the systems. The c(M) algorithm in SEDFIT-MSTAR was used to determine the baseline, as well as an estimated distribution of species. For lower concentrations and lower speeds, the software was unable to resolve individual components, but was able to provide point-average, apparent molar masses along the length of the cell (Fig. 7a). The 7 concentrations overlap in a range between 0 and 20 fringes. As would be expected, higher loading concentration samples showed higher concentration ranges. Apparent molar masses also ranged between 20 and 50 kDa. Considering the monomer molar mass of IGlu is 5.8 kDa, the hexameric mass is therefore 34.8 kDa. This suggests the distribution of components spans beyond hexamer to potentially di-hexamer assemblies. Such stoichiometries are not uncommon with insulin^4,17^. This is confirmed in the c(M) distribution analysis (Fig. 7b) where a peak is resolved at ~ 30 kDa (an underestimate of a hexamer due to the presence of non-ideality) and another peak at ~ 60 kDa (di-hexamer). The smaller peak resolved in AUC-SV is not present, however this may be due to the low concentration of the species. In the 22k revolutions per minute (RPM) sub-plot, one can clearly see the increase in apparent molar mass with decreasing concentration, as well as the decreasing concentration (from area-under-curve).

In summary, results from analytical ultracentrifugation confirm the presence of dimers, hexamers and di-hexamers, with the latter two in dynamic equilibrium.

## Conclusions

Glulisine (APIDRA) is a rapid-acting human insulin analogue indicated to improve glycaemic control in adults and paediatric patients, who present with diabetes mellitus, a condition which results from metabolic aberrations. The structural changes of IGlu, namely the replacement of the amino acid asparagine at position B3 by lysine and the replacement of lysine in position B29 by glutamic acid, are what effects the rapid-acting nature of this insulin. However, there are concerns as to the adverse effects of IGlu and therefore, more detailed analysis was required.

X-ray crystal structure analysis has revealed structural changes compared with insulin and other insulin analogues that can explain its rapid-acting role. This includes: firstly, the AsnB3Lys mutation, shows electrostatic repulsion to a neighbouring B22Arg. Secondly, the shorter residue Glu in the LysB29Glu mutation leads to fewer interactions to a symmetry related molecule (only one weak hydrogen bond to one of the D22Arg conformations compared to two to three hydrogen bonds in native insulin PDB 4EY9 and 4INS respectively, Fig. 4). Thirdly, the unique conformation of D21Glu now points to the core of the protein rather than outside towards the solvent, increasing dimer interactions, “pulling” the C-terminus closer so that D29Glu could make a hydrogen bond to the C1Gly N atom. These results are in agreement with AUC-SV analysis showing IGlu in solution as dimers. For the higher forms (hexamers and dihexamers) it could be a result of the low concentrations of Zn in solution and some interactions of IGlu with neighbouring molecules. These results show that the monomer to monomer interactions are weaker compared to native insulin. This can explain its fast dissociation to dimers and monomers and thereby its function as a rapid-acting insulin.

For the first time, this research provides novel, structural information on glulisine. This information sheds light on the dissociation of glulisine in solution which may be important for optimising drug formulation and reducing side effects of this drug. Knowledge of the structure could also be important when considering the docking/binding of glulisine with its receptor.

## Materials and methods

### Glulisine

10 mL vials of Insulin glulisine (IGlu) were produced by Sanofi Aventis and obtained from a pharmacy in Nottingham, United Kingdom. Vials were refrigerated at 5 °C until used.

### X-ray crystallography

#### Crystallisation

All crystallisation experiments were performed by the hanging-drop vapour-diffusion method by mixing the IGlu solution and the crystallisation agents at 1:1 ratio. Initial conditions were found using the commercial Index-I screen (Hampton Research) and construction of phase diagrams^10^. The crystallisation conditions were further refined by optimizing pH, precipitant concentrations and cryo parameters.

#### Data collection

Crystals were frozen straight from the original drop into liquid nitrogen. X-ray data was collected at 100K on the I04-1 beamline at Diamond Light Source synchrotron (λ = 0.91587 Å). The Pilatus 6M-F (active area of 424 × 435 mm^2^) detector operates in shutterless mode, allowing the collection of a complete dataset in a few minutes. A total of 900 frames were collected with oscillation range of 0.2° to 180°. Data processing was done automatically by the Diamond Xia2 software using mosflm for processing and aimless for scaling^18^.

A total of 104,577 accepted reflections (F > 0σ(F)) were measured in the 41.22–1.26 Å resolution range, and resulted in 22,987 independent reflections, with 99.8% completeness to resolution range and 99.3% completeness for the highest resolution shell of 3.42–1.26 Å. The overall redundancy in the data set was 4.5, the overall mosaicity was 0.066, the average $\left\langle {{\text{I}}/\sigma ({\text{I}})} \right\rangle$ was 13.4, and the final Rmerge (I +/−) for the whole data was 5.8% (69.1% for the highest resolution shell).

#### Structure determination of glulisine

The structure was solved by molecular replacement using Phaser^19^ and human insulin crystal structure (PDB entry: 4EY9) as search model, resulting in a clear solution Top LLG: 1149.97 and Top TFZ: 28.9 (RVAL: 45.1) parameter values and a dimer in the asymmetric unit. This conclusion was supported by the Matthews coefficient calculations^20^, which revealed that there are two molecules in the asymmetric unit, with a reasonable Vm value of 1.89 Å^3^/Da and estimated solvent content of about 35%. The structure was further refined using the Phenix software package^21^.

#### Model building and refinement of glulisine

Real space refinement of the IGlu dimer molecules was conducted by visual inspection of both the map and model with COOT^14^, which was also used for the addition of formate and zinc ions.

Each refinement cycle was followed by manual fitting and rebuilding of the two molecules of the IGlu dimer (chains A, B for Mol1 and C, D for Mol2) using COOT.

Further isotropic refinement was carried out with the program PHENIX. Water molecules were added at the end of refinement using the automated method provided in PHENIX while formate and Zn ions were added using COOT program. The model was further improved by anisotropic temperature factor refinement in PHENIX yielding *R*_work_ and *R*_free_ values of 12.79 and 15.18% (for 5% of the data), respectively.

#### Quality of final models

The stereochemical quality of the model was examined with the program PROCHECK^22^, allowing a general validation of its main structural parameters and as such is of high quality; see Table 1.

#### Calculations and figure preparations

The matrices for the superposition of the insulin and IGlu structures were calculated by a least-squares distance minimization algorithm (LSQ, implemented within the program PDBe fold), using the C α-atoms as the guide coordinates. Figures were prepared by the "Chimera" software^23^, COOT^14^ for electron density and electrostatic surfaces.

### Analytical ultracentrifugation

#### Sedimentation velocity

IGlu was assayed using a Beckman Optima XL-I analytical ultracentrifuge (Beckman Coulter, Brea, CA, USA). Data were acquired with ProteomeLab 6.0 from Rayleigh interference optical scans.

For concentrations ranges, IGlu preparations were concentrated using Centrisart tubes as to separate the protein from the excipient-only supernatant. IGlu vials were available at a concentration of 3.5 mg/mL, and subsequently diluted, using supernatant, into 6 concentrations (0.5, 1.0, 1.5, 2.0, 2.5, 3.0).

Sedimentation velocity (SV) experiments were performed at 45 k rpm (~ 150 kg) in duplicate. 400 μL of sample and reference were injected into AUC cells constructed from two-channel, aluminium-epoxy resin centrepieces, sapphire windows, and aluminium housings. Cells were balanced and aligned in the rotor (An50Ti) to match the direction of centrifugal force. Scans were performed every two minutes for 10 h.

SV data were analysed using SEDFIT v15.4. Continuous distribution of sedimentation coefficients analysis, c(*s*) vs *s*, was performed to identify peaks^24^. Peaks were integrated to calculate the absolute and relative concentration *c*, *%*, respectively. Values were corrected to standard conditions (water, 20 °C) to correct for density *ρ* and viscosity *η* variations (Eq. 1). A concentration series was also performed to calculate *k*_*s*_ according to Eq. (2).

1 $$s_{T,b} = s_{20,w} \left( {\frac{{(1 - \overline{v}\rho )_{20,w} }}{{(1 - \overline{v}\rho )_{T,b} }}} \right)\left( {\frac{{\eta_{T,b} }}{{\eta_{20,w} }}} \right)$$

2 $$s_{20,w} = s_{20,w}^{0} (1 - k_{s} c)$$

where *s* is the sedimentation coefficient, in temperature and buffer conditions _T,b_, extrapolated to infinite dilution ^*0*^.

#### HYDROPRO

Hydropro^25^ was used to estimate the sedimentation coefficient based on the crystal structure (from the PDB file) from a bead model of atomic positions. Partial specific volume was 0.729 mL/g and molar mass was 11,646 Da.

#### Sedimentation equilibrium

Sedimentation equilibrium (SE) experiments were performed on the same centrifuge as sedimentation velocity, except at 15k, 18k and 22 krpm. The same cells and rotor, as described above, were used but with 120 μL of sample and reference used. Scans were performed every hour until equilibrium was achieved i.e. no net movement of macromolecular material (~ 24 h).

Data were analysed using SEDFIT-MSTAR^26^. SEDFIT-MSTAR was capable of yielding weight-average, z-average molar masses, and low-resolution c(*M*) distributions (*c* = area-under-curve) from SE curves.

## Supplementary Information

Supplementary Information.
