# Supplementary material for: Analysis of insulin glulisine at the molecular level by X-ray crystallography and biophysical techniques
Source: Sci Rep. 2021 Jan 18;11:1737. doi: 10.1038/s41598-021-81251-2 (PMC7814034; doi:10.1038/s41598-021-81251-2)
Supplement: Supplementary file 1 — Supplementary Information. [file 41598_2021_81251_MOESM1_ESM.docx]

# **Analysis of insulin glulisine at the molecular level by X-ray crystallography and biophysical techniques**

**Supplementary Information**

Richard B. Gillis^1^*^, Hodaya V. Solomon^2^^, Lata Govada^2^, Neil J. Oldham^3^, Vlad Dinu^4^, Shahwar Imran Jiwani^1^, Philemon Gyasi-Antwi^1^, Frank Coffey^1^, Andy Meal^1^, Paul S. Morgan^1^, Stephen E. Harding^4,5^, John R. Helliwell^6^, Naomi E. Chayen^2^*, Gary G. Adams^1^*

^1^ University of Nottingham, Faculty of Medicine and Health Sciences, Queen’s Medical Centre, Nottingham, NG7 2HA, UK

^2^ Imperial College London, Biomolecular Medicine, Faculty of Medicine, South Kensington Campus, London, SW7 2AZ, UK

^3^ University of Nottingham, School of Chemistry, University Park, Nottingham, NG7 2RD, UK

^4^ University of Nottingham, National Centre for Macromolecular Hydrodynamics, School of Biosciences, Sutton Bonington Campus, Loughborough, LE12 5RD, UK

^5^ Universitetet I Oslo, Postboks 6762, St. Olavs plass, 0130 Oslo, Norway

^6^ University of Manchester, Department of Chemistry, Manchester, M13 9PL, UK

^ Richard B Gillis and Hodaya V Solomon are joint first Authors

*Co-corresponding authors:

[richard.gillis@nottingham.ac.uk](mailto:richard.gillis@nottingham.ac.uk)

n.chayen@imperial.ac.uk

[gary.adams@nottingham.ac.uk](mailto:gary.adams@nottingham.ac.uk)

Figure S1: ESI-MS trace of glulisine sample, zoomed into m/z 1164-1167. Upper trace: experimentally obtained result. Lower trace: theoretical predicted ions based on the formula of glulisine provided. There is good agreement between isotope patterns, thus confirming the presence of IGlu in the samples analysed further through x-ray crystallography and biophysical techniques.


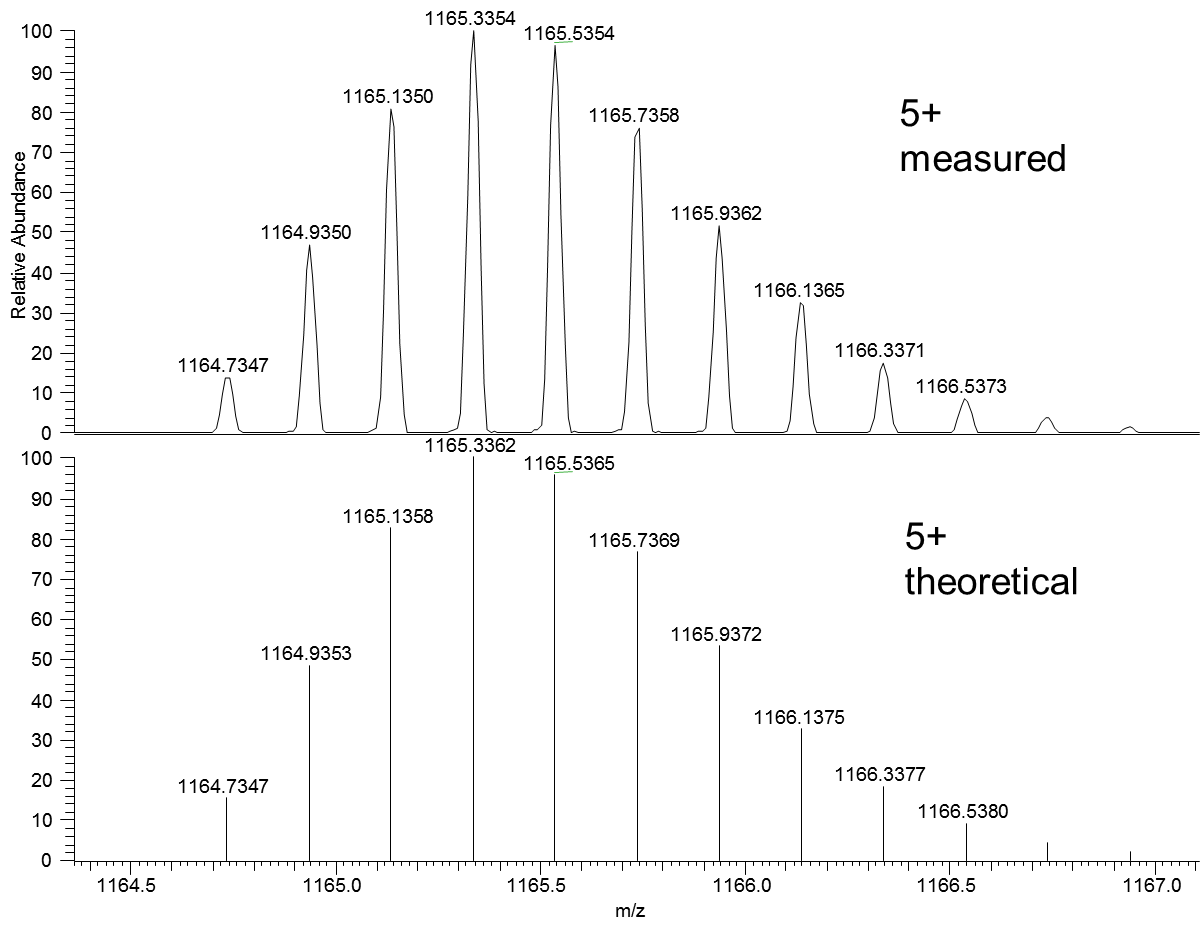


**Mass Spectrometry Results**

To confirm the constituents of the sample, mass spectrometry was employed with soft-ionisation. The electrospray ionisation-mass spectrum (ESI-MS) of denatured IGlu exhibited charged states from [M+3H]3+ to [M+6H]6+. Deconvolution of the spectrum gave a measured mass of 5818.6364 Da for the peptide, which was in excellent agreement with the predicted monoisotopic mass of IGlu (5818.6373 Da; 0.16 ppm mass error) (Figure S1), with its associated Lys residue at position B3 and Glu at position B29. To further confirm the identity of the peptide, MS/MS fragmentation was performed on the [M+5H]5+ ion. Fragment ions corresponding to the loss of Thr (5699.58), Pro-Glu-Thr (5473.49) and Thr-Pro-Glu-Thr (5372.44) were all visible confirming the presence of Thr at position 30, and Glu at position 29 in the B-chain.

**Mass Spectrometry Materials and Methods**

A 10μL sample of IGlu (APIDRA, Sanofi-Aventis, Reading, UK) was desalted using C18 ZipTip^TM^ (Merck, Watford, UK) solid phase extraction following the manufacturer’s instructions. Briefly, the sample solution was loaded onto the tip by five 10μL aspirate/dispense cycles, washed ten-times with 10μL water (18 MOhm) containing 0.1% formic acid, and eluted with 10μL 80:20 acetonitrile water (0.1 % formic acid). The solution was diluted to 100 μL with the same solvent and a 10 μL aliquot loaded into a nanoelectrospray tip (prepared in house using a Sutter P97 micropipette puller) connected to a platinum wire electrode.

Mass spectra were acquired on a ThermoFisher (Waltham, MA, USA) LTQ FT Ultra mass spectrometer (a hybrid linear ion trap-Fourier transform ion cyclotron resonance (FTICR) instrument equipped with a 7 Tesla magnet. The nanoelectrospray (nESI) source was operated at 1.5 kV. The inlet capillary of the mass spectrometer was held at 275 °C with a tube lens value of 145 V. Mass spectra were measured at a resolving power of 100000 (quoted at m/z 400), and MS/MS spectra were generated using collision induced dissociation (CID) in the ion trap of the instrument using helium as a buffer gas and a nominal collision energy of 35. Data were analysed using Xcalibur software (ThermoFisher).

Figure S2: A typical IGlu crystal


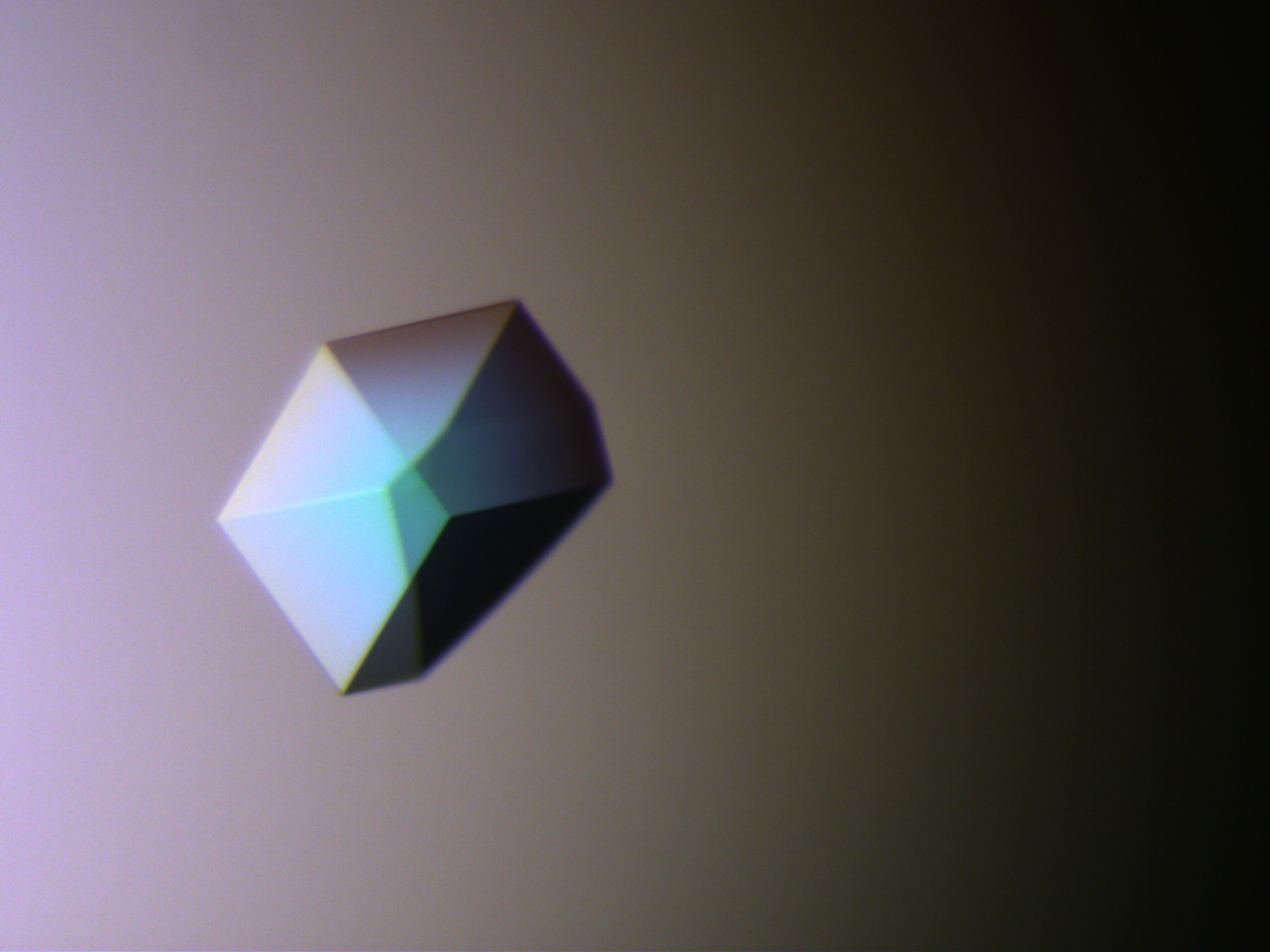


Figure S3: Electron density of glulisine a. example of the clear highly resolved structure of the aromatic residues of the B chain β-strand (2σ). b. electron density for D29Glu mutant residue, H-bonded to C1Gly N atom (1σ). c. well defined electron density of D3Lys stabilised by two H-bonds to water and FMT molecules. Figures created using COOT v0.8.9.1


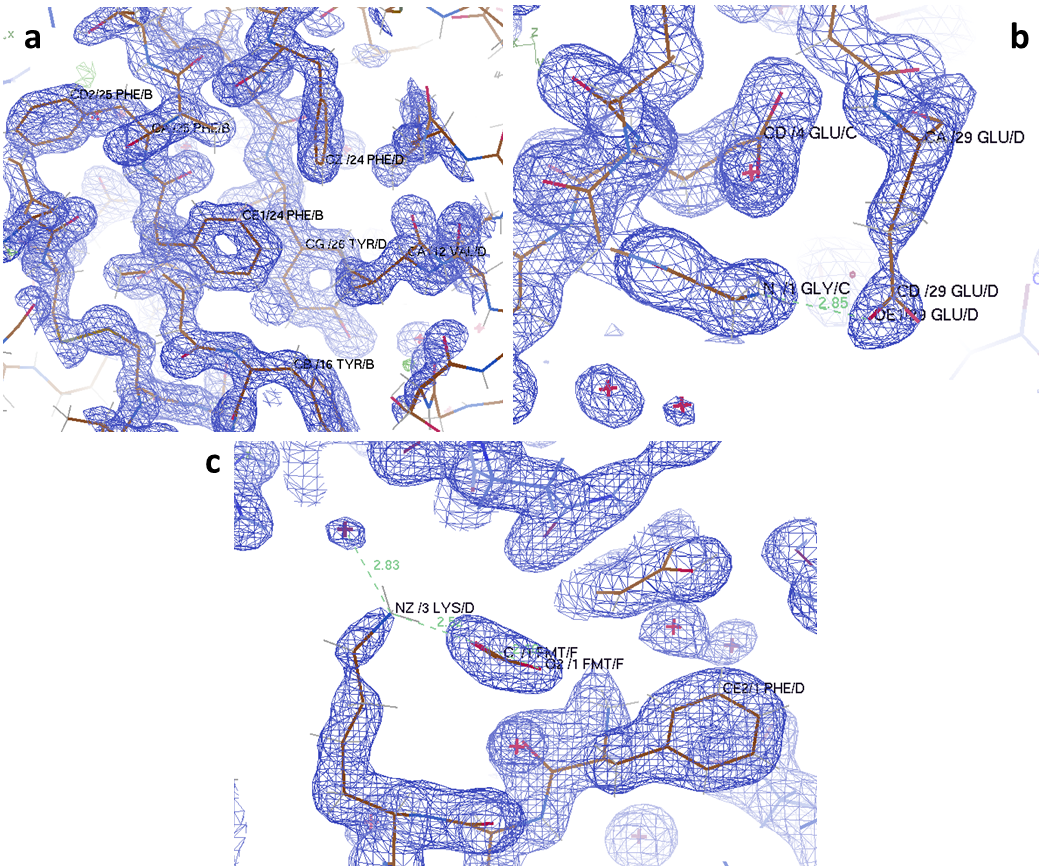


Figure S4: Three fold symmetry axis along the zinc ions (purple). Chloride ion in green, IGlu in tan, 4INS in light blue and 4EY9 in red. Figure created using Chimera v1.8.1 www.cgl.ucsf.edu/chimera/


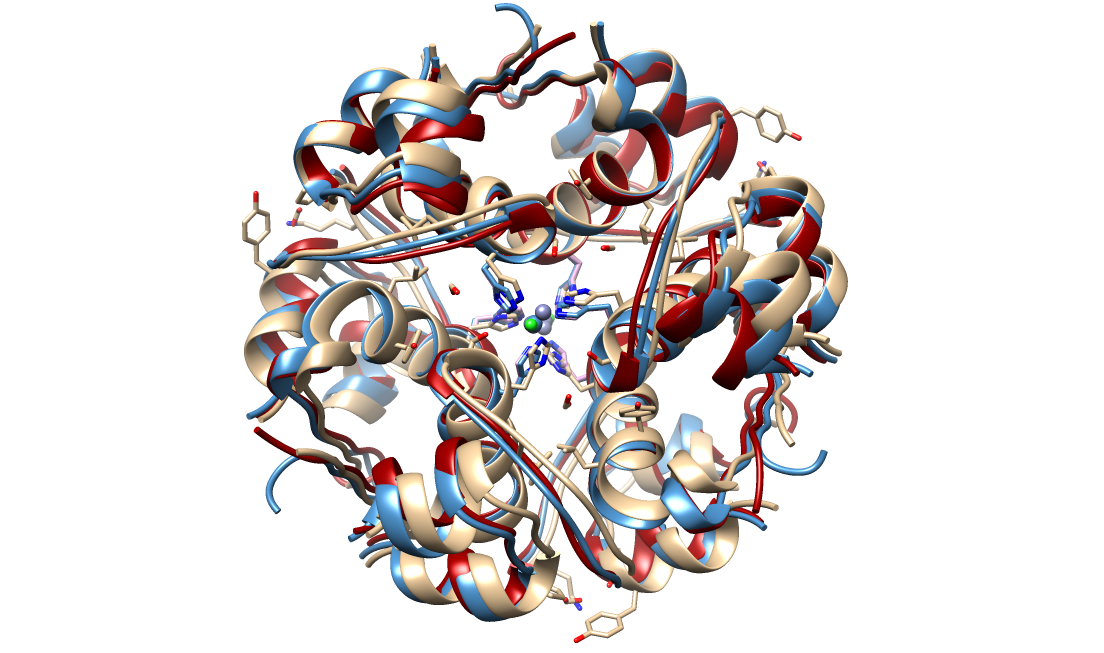


Table S1: RMSD values of IGlu chains superimposed with 4EY9 and 4INS insulin analogues

| Glulisine  Chain | 4EY9  Chain (rmsd) | 4INS  Chain (rmsd) |
| --- | --- | --- |
| A | A 0.45 | A 1.08 |
| B | B 0.36 | B 0.61 |
| C | C 0.34 | C 1.06 |
| D | D 0.57 | D 0.64 |
| A | C 1.06 | C 0.36 |
| D | B 0.74 | B 0.43 |
